# Histogram-based normalization technique on human brain magnetic resonance images from different acquisitions

**DOI:** 10.1186/s12938-015-0064-y

**Published:** 2015-07-28

**Authors:** Xiaofei Sun, Lin Shi, Yishan Luo, Wei Yang, Hongpeng Li, Peipeng Liang, Kuncheng Li, Vincent C T Mok, Winnie C W Chu, Defeng Wang

**Affiliations:** Department of Imaging and Interventional Radiology, The Chinese University of Hong Kong, Shatin, New Territories, Hong Kong, China; Research Center for Medical Image Computing, The Chinese University of Hong Kong, Shatin, New Territories, Hong Kong, China; Department of Biomedical Engineering and Shun Hing Institute of Advanced Engineering, The Chinese University of Hong Kong, Shatin, New Territories, Hong Kong, China; Department of Medicine and Therapeutics, The Chinese University of Hong Kong, Shatin, New Territories, Hong Kong, China; Lui Che Woo Institute of Innovation Medicine, The Chinese University of Hong Kong, Shatin, New Territories, Hong Kong, China; Shenzhen Research Institute, The Chinese University of Hong Kong, Shenzhen, China; School of Geoscience and Info-Physics, Central South University, Changsha, China; Department of Radiology, The Second Hospital of Jilin University, Changchun, Jilin China; Department of Radiology, Xuanwu Hospital, Capital Medical University, Beijing, China

**Keywords:** Magnetic resonance imaging, Intensity normalization, Histogram normalization, Noise estimator, Brain template construction

## Abstract

**Background:**

Intensity normalization is an important preprocessing step in brain magnetic resonance image (MRI) analysis. During MR image acquisition, different scanners or parameters would be used for scanning different subjects or the same subject at a different time, which may result in large intensity variations. This intensity variation will greatly undermine the performance of subsequent MRI processing and population analysis, such as image registration, segmentation, and tissue volume measurement.

**Methods:**

In this work, we proposed a new histogram normalization method to reduce the intensity variation between MRIs obtained from different acquisitions. In our experiment, we scanned each subject twice on two different scanners using different imaging parameters. With noise estimation, the image with lower noise level was determined and treated as the high-quality reference image. Then the histogram of the low-quality image was normalized to the histogram of the high-quality image. The normalization algorithm includes two main steps: (1) intensity scaling (IS), where, for the high-quality reference image, the intensities of the image are first rescaled to a range between the low intensity region (LIR) value and the high intensity region (HIR) value; and (2) histogram normalization (HN),where the histogram of low-quality image as input image is stretched to match the histogram of the reference image, so that the intensity range in the normalized image will also lie between LIR and HIR.

**Results:**

We performed three sets of experiments to evaluate the proposed method, i.e., image registration, segmentation, and tissue volume measurement, and compared this with the existing intensity normalization method. It is then possible to validate that our histogram normalization framework can achieve better results in all the experiments. It is also demonstrated that the brain template with normalization preprocessing is of higher quality than the template with no normalization processing.

**Conclusions:**

We have proposed a histogram-based MRI intensity normalization method. The method can normalize scans which were acquired on different MRI units. We have validated that the method can greatly improve the image analysis performance. Furthermore, it is demonstrated that with the help of our normalization method, we can create a higher quality Chinese brain template.

## Background

Magnetic resonance imaging (MRI), as a non-invasive imaging method, has been widely used to study and analyze human brains. Although lacking of a normalized intensity scale of MRI has no direct effect on clinical medical diagnosis by doctors, the situation is complicated by some image post-processing technique, such as automatic segmentation, registration and quantification method, which are highly dependent on the intensity information to achieve favorable results. In particular, for the large-scale multi-site neuroimaging studies involving a significant number of subjects scanned with different scanner types and scanning parameters. The differences in subject positioning between sites or a baseline and a later scan, or protocol can be found, making the interpretation difficult without intensity normalization. Normalization of the observed image intensities is of crucial importance to explore the disease progression in many clinical studies. However, images from different scanners or with different acquisition parameters may have large intensity variations, which greatly affects the results of image analysis. Therefore, an intensity normalization of MRI scans, which aims at correcting for scanner-dependent variations, is essential for accurate MRI analysis. The previous studies concerning work on MRI intensity normalization are briefly reviewed as follows.

A histogram matching method was proposed for correcting the variations in scanner sensitivity due to differences in scanner performance [1]. It was shown that this method can reduce the variations in white matter (WM) intensities from 7.5 to 2.5%. Furthermore, the utility of even order derivative analysis in the MRI histogram was demonstrated in [2]. It was shown that good WM peak discrimination can be achieved even when there is a large overlap between gray matter (GM) and WM peaks, as is the case with the T2-weighted brain images. Furthermore, the ability of the normalization procedure to correct the global intensity variations over time was demonstrated by the high degree of reproducibility in segmentation results. In another study [3], an image post processing method was proposed for integrating multiple serial MRI scans into a volume to facilitate quantitative evaluation of the temporal intensity profiles. A fatal error reduction was observed when applying tissue specific inter-scan intensity normalization. Nyul et al. proposed a method consisting of a training stage to find the parameters of the standard scale and a transformation stage to map the histograms of candidate volumes onto the standard histogram scale [4]. The effectiveness of the above method proposed in [5], was later evaluated in [6] for rendering. It was demonstrated that the lesion segmentation result is more accurate after applying the normalization method. The above intensity normalization algorithms are mainly designed to align MRI intensities to a standard grayscale. In addition, some histogram matching algorithms were designed to match the histogram of the input image with the histogram of the reference image by minimizing some information-centric criteria, such as through a joint histogram [7]. But this method suffers from unreliable processing results [8]. Because, in order for the existing histogram matching based on a joint histogram to achieve a more reliable implementation, it required a better prior knowledge based of the neighborhoods used to split up the image into K sub-images, which are corrected separately [9]. However, this method relies on a non-rigid registration to match the histogram, making it considerably slower than approaches which normalize an image without using additional preprocessing steps. As it is desired that intensity normalization as an image preprocessing method should be efficient and easy to implement, we propose a simple global histogram normalization method, which has a low computation complexity and no parameter tuning.

In this paper, we propose to implement intensity normalization to achieve homogeneous intensities or similar image quality for two brain MR images acquired from two different field strength scanners with different acquisition parameters. Firstly, a noise estimation model was employed to assess the image quality. Secondly, the high-quality image served a as reference and was first preprocessed with intensity scaling. Finally, histogram normalization was implemented on the low-quality image to match the reference histogram of the preprocessed high-quality image. During MR image acquisition, different machines or parameters would be used for scanning different subjects or the same subject at a different time, which may result in large intensity variations across scans. Our normalization method can be applied in many situations. For example, in population analysis, our method is essential for normalizing intensity level in different subjects and making consistent analysis results. In addition, to analyze one subject’s MRIs scanned at different time, our method can be helpful for eliminating the effect of imaging environment and parameters change. In our experiments, the performance of our method was qualitatively and quantitatively evaluated with three image analysis experiments, i.e., image registration, image segmentation and tissue volume measurement. We have validated that the intensity normalization method can help improve low-quality images and thus increase the accuracy of the image analysis result. During MR image acquisition, different machines or parameters would be used for scanning different subjects or the same subject at a different time, which may result in large intensity variations across scans. Our normalization method may be applied in many cases. For the MR images of different scanning subjects, the most high-quality MR image from the same dataset or a standard high-quality brain template image served as the reference through image quality estimation. For MR images of the same subject at a different time, the high-quality MR image from the same subject served as the reference through image quality estimation to perform intensity normalization upon with respect to the same subject. In our method, we always guarantee the high-quality image to be provided as the reference image in order to maintain the overall high quality of normalized images. To popularize our method, we also employed this normalization technique as a preprocessing step to construct Chinese brain template for a group of subjects aging from 20 to 30 years, and studied the effectiveness of intensity normalization on the quality of Chinese brain template in large-scale image dataset.

## Methods

### MRI data acquisition and preprocessing

The study was approved by the Ethics Committee of the Chinese University of Hong Kong at the Prince of Wales Hospital. All the selected subjects signed consent forms. In this study, the brain MRI images used for experimental evaluation consists of 22 images, two for each patient, which were acquired from the Department of Imaging and Interventional Radiology, Prince of Wales Hospital in Hong Kong, China during July 2011. Eleven adult subjects (4 males, and 7 females), with ages ranging from 20 to 70 years old were scanned twice with a Siemens Sonata (Siemens Medical System, Iselin, NJ) 1.5T MRI scanner and a Philips Achieva (Philips Medical Systems, Best, the Netherlands) 3.0T MRI scanner, with an interval of 8–12 min. The sagittal MR images used for analysis were obtained using a T1-weighted fast field echo (FFE) pulse sequence (repetition time = 25 ms, echo time = 2.36 ms, flip angle = 30) when using the 1.5T scanner, and a T1-weighted inversion recovery multiplanar reformatting (IR-MPR) pulse sequence (repetition time = 1,990 ms, echo time = 3.93 ms, inversion time = 1,100 ms) when using the 3.0T scanner. The reconstructed images of the 3T unit had a slice thickness of 1.8 mm and a field of view of 230 mm with a pixel resolution of 1.239 pixels per mm and a 256 × 256 matrix. And the images of the 1.5T unit had a slice thickness of 1 mm and a field of view of 230 mm with a pixel resolution of 4.956 pixels per mm and a 256 × 216 matrix. A built-in radiofrequency (RF) body coil within the 1.5T MRI scanner and dual RF surface coils with a parallel reconstruction scheme within 3.0T MRI scanner were used for the radio signal transmission and signal detection, respectively.

The intensities and quality of the images with different parameters were quite different. As the intensity percentage of the skull dramatically varies for different settings; brain extraction was performed to obtain a robust normalization result. The Brain Extraction Tool (BET) of the FSL (FMRIB’s Software Library) software is a fast robust automated tool for skull stripping [10]. This tool was utilized to remove non-brain areas. For complete brain stripping, we have to manually modify the results produced by this tool, as an automated extraction may not eliminate the skull well enough; which can lead to later errors in registration and segmentation.

### Image quality assessment

As the images were acquired from different scanners with various acquisition parameters (e.g. repetition time, echo time, or flip angle), the quality of the scanned images was very different. The objective of our study was to normalize the histogram of a low-quality MRI to the histogram of a high-quality MRI in order to improve the image quality of the low-quality MRI. Therefore, it is crucial to assess the quality of the images first. Numerous approaches have been proposed for MRI quality assessment [11]. As our intensity normalization method aims to normalize histograms, we will use a histogram to assess the image quality. We employed the noise estimation method proposed by Aja-Fernández et al. [12, 13]. It uses the histogram of MRI based on the background intensity only and does not need any prior knowledge. The Aja-Fernández’s estimator assumes a Rayleigh probability density function (PDF) in the background of the image [12, 13]. The estimation was done without segmentation, by taking the maximum value of some local distribution. It defines the noise estimation index $$ \overset{\lower0.5em\hbox{$\smash{\scriptscriptstyle\frown}$}}{\sigma }_{n} $$ as,

1 $$ \overset{\lower0.5em\hbox{$\smash{\scriptscriptstyle\frown}$}}{\sigma }_{n} = \text{mode} \{ M(x)\} $$

where $$ \text{mode} \{ M(x)\} $$ is a mode of the distribution of image $$ M(x) $$, and $$ \overset{\lower0.5em\hbox{$\smash{\scriptscriptstyle\frown}$}}{\sigma }_{n} $$ is a noise estimation index. With the calculated index, the MRI quality can be directly assessed. A smaller value of the estimation index indicates a higher quality of the image.

### Histogram-based normalization

A normalization algorithm adjusts the distributions of each follow up scan to match the chosen baseline scan in order to improve image similarity and facilitate MR image comparability between MRI scans [3]. As mentioned previously, the Nyul’s algorithm [5] of intensity normalization relies on landmarks to achieve normalization. However, reliable landmarks are usually difficult to consistently locate [14]. Therefore, we propose the following histogram normalization algorithm without requiring any prior knowledge or manual intervention.

For the same subject, the proportions of intensity levels for the same tissue type for brain MRIs from scanners with different field strengths and various acquisition parameters were similar. Thus, a high-quality image serves as a reference image, and the low-quality one will be serve as the input image. The tails of the histogram often cause problems. Usually the high intensity tail corresponds to artifacts and outlier intensities, which causes considerable scanner variations. To avoid this problem, we first preprocess the reference image, removing the background and outliers, which results in the intensity of interest (IOI) as the standard scale.

Our overall approach includes two steps as follows. Denote the minimum and the maximum intensities on the standard scale (corresponding to the standard histogram) as $S_{\rm{min} }$ and $S_{\rm{max} }$.

1. Intensity scaling (IS). For the reference image, the histogram is composed of homogeneous low intensity regions of interest (low intensity region-LIR) and high intensity regions of interest (high intensity region-HIR).The histogram starts at LIR and extends up to HIR brightness levels. The image intensities are mapped to the values between HIR and LIR. Here, HIR is defined as the value at the maximum decile, and LIR is defined as the value at the minimum decile. These definitions can help remove background noise and outliers as described above. It is implemented as follows:
  2 $$ f^{\prime}(x,y,z) = \frac{f(x,y,z) - LIR}{HIR - LIR} $$
  where $f(x,y,z)$ is a gray value of original reference at $(x,y,z)$, and $f^{\prime}(x,y,z)$ is the corresponding transformed grayscale value.
2. Histogram normalization (HN). The reference image histogram is stretched, and shifted in order to cover all the grayscale levels in the input image as follows,
  3 $$ g^{\prime}(x,y,z) = \frac{HIR - LIR}{{S_{\rm{max} } - S_{\rm{min} } }}\left( {g(x,y,z) - S_{\rm{min} } } \right) + LIR $$

If the target histogram of the input image $g(x,y,z)$ starts at $S_{\rm{min} }$ and extends up to $S_{\rm{max} }$ grayscale levels in IOI, then we can scale up the image between the lower boundary $m_{1}^{\prime }$ and the upper boundary $m_{2}^{\prime }$ so that the voxels in the new normalized image $g^{\prime}(x,y,z)$, will lie between a minimum level (*LIR*) and a maximum level (*HIR*). The following variables $m_{1}$ and $m_{2}$ are the lower boundary and upper boundary of the reference image prior to scaling up, respectively.

**Fig. 1:**
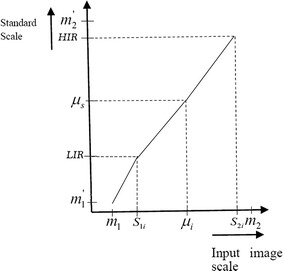
The intensity normalization function for the histogram-based normalization phase. The first mapping is from [$S_{1i}$, $\mu_{i}$] to [$LIR$, $\mu_{s}$] and the second is from [$\mu_{i}$, $S_{2i}$] to [$\mu_{s}$, $HIR$]. The *lower* and the *upper* ends of the standard scale are subsequently extended to $m^{\prime}_{1}$ and $m^{\prime}_{2}$, respectively, by mapping [$m_{1}$, $S_{1i}$] to [$m^{\prime}_{1}$, $LIR$] and [$S_{2i}$, $m_{2}$] to [$HIR$, $m^{\prime}_{2}$].

This is done by doing two separate linear mappings. The first is from [$S_{1i}$, $\mu_{i}$] to [$LIR$, $\mu_{s}$] and the second is from [$\mu_{i}$, $S_{2i}$] to [$\mu_{s}$, $HIR$]. Figure 1 shows the plot of the mapping function. The lower and the upper ends of the standard scale are subsequently extended to $m^{\prime}_{1}$ and $m^{\prime}_{2}$, respectively, by mapping [$m_{1}$, $S_{1i}$] to [$m^{\prime}_{1}$, $LIR$] and [$S_{2i}$, $m_{2}$] to [$HIR$, $m^{\prime}_{2}$], as illustrated in Fig. 1. We call this mapping from the intensities [$m^{\prime}_{1}$, $m^{\prime}_{2}$] to [$m_{1}$, $m_{2}$] of the standard scale the normalization of the input image. We denote the normalization function as $N(x,y,z)$. The expression for $N(x,y,z)$ (from Fig. 1) is:

4 $$ N(x,y,z) = \left\{ \begin{aligned} \left\lceil {\mu_{s} + (g(x,y,z) - \mu_{i} )\frac{{LIR - \mu_{s} }}{{S_{1i} - \mu_{i} }}} \right\rceil ,\quad m^{\prime}_{1} \le g(x,y,z) \le \mu_{i} \hfill \\ \left\lceil {\mu_{s} + (g(x,y,z) - \mu_{i} )\frac{{HIR - \mu_{s} }}{{S_{2i} - \mu_{i} }}} \right\rceil ,\quad \mu_{i} \le g(x,y,z) \le m^{\prime}_{2} \hfill \\ \end{aligned} \right. $$

where $\left\lceil \bullet \right\rceil$ denotes the ‘‘ceiling’’ operator, $\mu_{i}$ and $\mu_{s}$ are the mean values for the input image histogram and reference image histogram, respectively. $S_{1i}$ and $S_{2i}$ are the voxel values from the input image. In this work, instead of using the suggested 10 percentile intensity landmarks of Ref. [4], we have used three important intensity values [minimum (e.g. *LIR* and $S_{1i}$), maximum (e.g. *HIR* and $S_{2i}$) and mean (e.g., $\mu_{i}$ and $\mu_{s}$)], which will be achieved easily without depending on unreliable landmarks in the histogram for normalizing the MRI images. Therefore, the proposed normalization method is not essentially the same as the existing normalization method.

After normalizing the input image (low-quality image) to the reference image (high-quality image), the performance of histogram normalization is estimated with noise estimation.

## Evaluation methodology and experiments

In these experiments, we have tried to incorporate as wide a range as possible to reflect variations in scanners, such as pulse sequence, magnetic field strength, and slice thickness in the MRIs utilized for this purpose. The evaluation of the normalization algorithm was performed to examine the accuracy improvement of MRI analysis after histogram-based normalization. The following three aspects are considered for evaluation.

1. Whether normalization helps in improving the accuracy of non-rigid registration compared to other normalization methods.
2. Whether automatic brain tissue [white matter (WM), gray matter (GM) and cerebrospinal fluid (CSF)] segmentation result is improved by using normalization.
3. Whether normalization helps in making volumes of different tissue in a normalized MR image more similar with volumes in high-quality MR images for the same subject after normalization.

Firstly, we performed non-rigid registration experiments to ascertain whether the normalization helps in increasing the non-rigid registration accuracy after normalization. We employed FSL FNIRT tool to perform non-rigid registration [15]. In our experiments, the nonlinear version of the MNI152 brain template is used as a reference image in registration. The original low-quality images, normalized low-quality images and high-quality images served as moving images and registered with the reference image. We compared our method with the histogram matching method based on the joint histograms algorithm [7]. The evaluation of both visual inspection and quantitative comparison were performed using a mean square error (MSE),

5 $$ MSE = \frac{1}{n}\sum\limits_{i = 1}^{n} {(\overset{\lower0.5em\hbox{$\smash{\scriptscriptstyle\frown}$}}{Y}_{i} - Y_{i} )^{2} } $$

where $$ \overset{\lower0.5em\hbox{$\smash{\scriptscriptstyle\frown}$}}{Y}_{i} $$ is the voxel intensity of the registered image, and $$ Y_{i} $$ is the corresponding voxel intensity of the reference image.

Secondly, a Markov random field segmentation algorithm [16] was applied to our MRI scans to segment three main tissues namely: WM, GM and CSF. We performed affine registration first to make the dimensions of all the images uniform. The results of tissue segmentation of the high-quality image were used as the gold standard for segmentation result comparison. The segmentation result was measured by a volume overlap using the Dice similarity coefficient (DSC) [17].

6 $$ DSC(X,Y) = \frac{{2\left| {X \cap Y} \right|}}{|X| + |Y|} $$

where X and Y represent binary label images for two compared segmentation images. DSC ranges from 0 to 1, with a higher DSC indicating a better overlap.

Finally, we utilized the three main tissue volumes measurement to evaluate our normalization method. To achieve this goal, we used the FreeSurfer tool (http://surfer.nmr.mgh.harvard.edu/) to count the volumes of WM, GM and CSF for each subject. And we compared the volume difference of all the three main tissues with tissue volumes of high-quality images for the same subject before and after normalization.

We validate our histogram normalization algorithm based on real brain MRI data. The existing MRI data consists of two sets of 22 T1-weighted MR brain images (obtained using 1.5T Siemens scanner with FFE sequence and 3T Philips scanner with IR-MPR sequence, respectively) from 11 subjects, that is to say, each subject had two images with different field strengths from each of the Philips and Siemens MRI scanners acquired.

Meanwhile, one important application of our intensity normalization method is to serve as a critical preprocessing step for Chinese brain template construction on young adults. In many template construction works [18–21], before brain template or atlas construction, preprocessing procedures of MR images are essential to improve the image quality. With intensity normalization, the boundaries between some brain tissues, such as WM, GM, and CSF, can be much clearer, helping increase the accuracy of ensuing image registration for template construction. Hereby, our proposed normalization technique on whole dataset was used as a preprocessing for Chinese brain template construction. We used MNI52_1 mm template, an average template of 152 T1 MRI scans of the same individual, as the reference brain scan for intensity normalization.

The test data were collected from 100 normal Chinese adult volunteers acquired using three different scanners, i.e. GE, SIEMEMS and PHILIPS. The age of the subjects ranges from 20 to 30 years (mean of 24.49 years). The dataset consists of 47 female and 53 male subjects. None of the subjects had any history of neurological, psychiatric, or significant medical illness. A set of T1-weighted brain images was acquired in the sagittal plane with a GE 3T Discovery MR750 scanner with an 8HRBRAIN coil. A 3D BRAVO sequence was applied with following parameters: 130 slices, TR/TE = 8.208/3.22 ms, flip angle = 12º, FOV = 256 × 256 mm^2^, matrix size = 256 × 256, slice thickness = 1 mm. Another set of T1-weighted brain images was acquired in the sagittal plane with a SIEMENS Verio 3T scanner with a body coil. A 3D tfi3d1 sequence was applied with following parameters: 176 slices, TR/TE = 1,900/2.5 ms, flip angle = 9º, FOV=218 × 250 mm^2^, matrix size = 384 × 336, slice thickness = 1 mm. The rest set of T1-weighted brain images was acquired in the sagittal plane with a Phillips Achieva 1.5 T MRI scanner with a head coil. A 3D FFE sequence was applied with following parameters: 301 slices, TR/TE = 25/4.6 ms, flip angle = 15º, FOV=256 × 256 mm^2^, matrix size = 256 × 256 slice thickness = 1 mm.

We used the procedures for constructing the brain template proposed in [18]. One in-house existing template image served as the reference image. All the images were first affinely registered to the reference image. An initial group mean image was generated and served as intermediate template image. Then nonrigid symmetric image normalization (SyN) algorithm in ANTS software (http://www.picsl.upenn.edu/ANTS/) is applied to perform a groupwise registration to bring the population of images into the common space. Cross-correlation (CC) is used as our similarity metric. Our brain template of the 100 Chinese young adults was generated by iteratively performing the above registration for three iterations.

## Results

Firstly, the efficiency of our intensity normalization is favorable in the aspect of runtime. On the runtime for normalizing a typical T1 structural brain MRI, our intensity normalization utilized piecewise linear transformation. So computational complexity of our intensity normalization is relatively lower, and the runtime (≈1 s) is shorter than the existing intensity normalization (runtime ≈40 s). The runtime measurements were performed on an Intel Xeon(R) CPU E5606 with 2.13 GHz and 16 gigabyte RAM.

At high noise levels, histogram matching may be dependent on the number of bins [14]. But in the experiments, the noise of images is eliminated to a great degree during the image acquisition. The number of bins may not negatively affect the results of intensity normalization and the post-processing steps. Then, we evaluated our results using three methods including registration, segmentation and three main tissues’ (e.g. WM, GM, and CSF) volumes measurement and compared these with the existing intensity normalization achieved by the histogram matching method. The performance of this normalization method was evaluated through the performance of automatic brain segmentation and also utilized as a preprocessing step for brain template construction.

### Registration

**Fig. 2:**
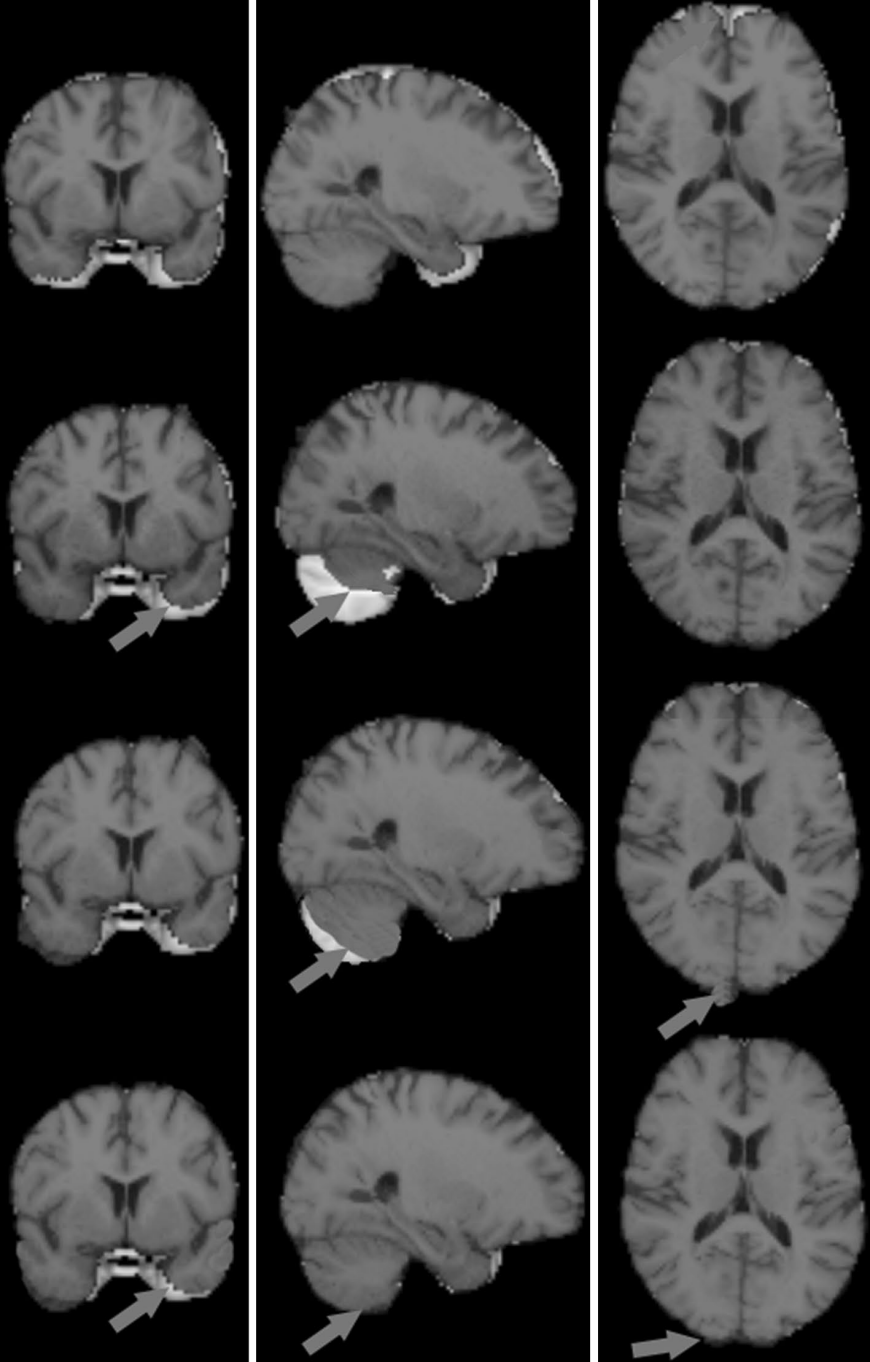
Visual results for the reference image, input image and two normalized images in the same slice for one subject registered with the brain template image MNI152-2mm_brain. From *top* to *bottom*: reference image, input image, and images normalized with Hist. Matching and Hist. Normalization overlaid with template image. From *left* to *right*: coronal slice, sagittal slice, axial slice. The *arrow* points to the differences of visual results of registration between images and brain template image.

**Fig. 3:**
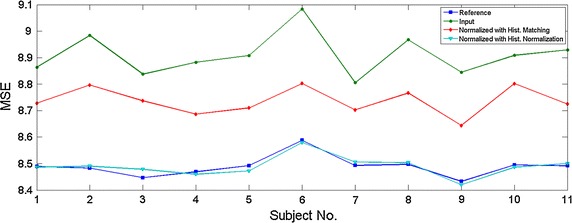
Mean square error (MSE) between all images and the template image (MNI152_2mm_brain) are shown for different subjects.

We present one example of results for a female subject scanned with the 1.5T and 3.0T machines, respectively. From Fig. 2 shows the non-rigid registration results of the low-quality image before and after normalization, as well as the high-quality image. The reference image is the T1-weighted MNI152_2mm_brain template. The MSEs between all the registered images and the reference images are shown in Fig. 3 and Table 1. From Fig. 3 and Table 1, we can observe that the MSE values with our normalized image are smallest among the four sets of MSE values, which validates that our histogram normalization is efficient in improving registration accuracy.

**Table 1:** The mean square error (MSE) between all images and the template image

Image type	MSE
Reference image	8.4887 ± 0.0393
Input image	9.0086 ± 0.3112
Normalized image with hist. matching	8.7366 ± 0.0516
Normalized image with hist. normalization	8.4892 ± 0.0385

Data layout: mean ± std.

### Segmentation

We report Dice similarity coefficients (DSC) in Table 2 between segmentations of the three brain main tissues, WM, GM, and CSF. The values are averaged over eleven subjects, each with two scans. Histogram normalization produces significantly higher DSC for GM and WM compared to histogram matching based on the minimization of some information-centric criteria, while the DSC indexes are comparable for the CSF segmentation. Use of the histogram matching method sometimes may even decrease the performance compared with the original image, as its performance is highly dependent on the number of histogram bins.

**Table 2:** Average dice coefficients of hard segmentations were obtained from 22 scans, before and after normalization, comparing our method with that of histogram matching [7]

	WM	GM	CSF	Mean
DSC (input image)	0.8092 ± 0.0190	0.6700 ± 0.0496	0.5225 ± 0.0351	0.6751
DSC (normalized image with hist. matching)	0.8149 ± 0.0196	0.6768 ± 0.0506	0.5309 ± 0.0358	0.6832
DSC (normalized image with hist. normalization)	0.8261* ± 0.0178	0.6884* ± 0.0494	0.5482* ± 0.0362	0.6986

Data layout: mean ± std.

* Statistically significantly larger than the other two (*p* value 0.05).

### Volumes estimation

We first compute three main tissue (WM, GM, and CSF) volumes for the un-normalized MRIs using the FreeSurfer software. For the same subjects, volumes of the same tissue (e.g. WM, GM, and CSF) for different images from the two scanners were very similar. Intensity boundaries between various tissues were blurry; after normalization the boundaries become relatively clear. We compare the volumes of WM, GM, and CSF, before and after normalization between the low-quality images and the high-quality images from the same subject, meanwhile, an additional comparison with the histogram matching method was performed. As shown in Table 3, it can be shown that the average volumes of WM, GM and CSF for the low-quality image with histogram normalization are closer to the high-quality image than the histogram matching method.

**Table 3:** Average tissues volumes of WM, GM and CSF (without sulcal CSF) obtained from 22 scans in the eleven subjects, before and after normalization, comparing our method with histogram matching (unit: mm^3^)

	WM	GM	CSF (without sulcal CSF)
Volume (reference image)	468,400* ± 54,664	538,990* ± 49,667	1,260* ± 193
Volume (input image)	409,480 ± 54,011	572,560 ± 47,994	1,058 ± 187
Volume (normalized image with hist. match)	443,790 ± 56,273	547,810 ± 53,457	1,148 ± 189
Volume (normalized image with hist. normalization)	451,083* ± 53,326	540,940* ± 48,757	1,225* ± 199

Data layout: mean ± std.

* Statistically significantly the volumes with HN method are identical to the volumes of the reference image (*p* value <0.05).

**Fig. 4:**
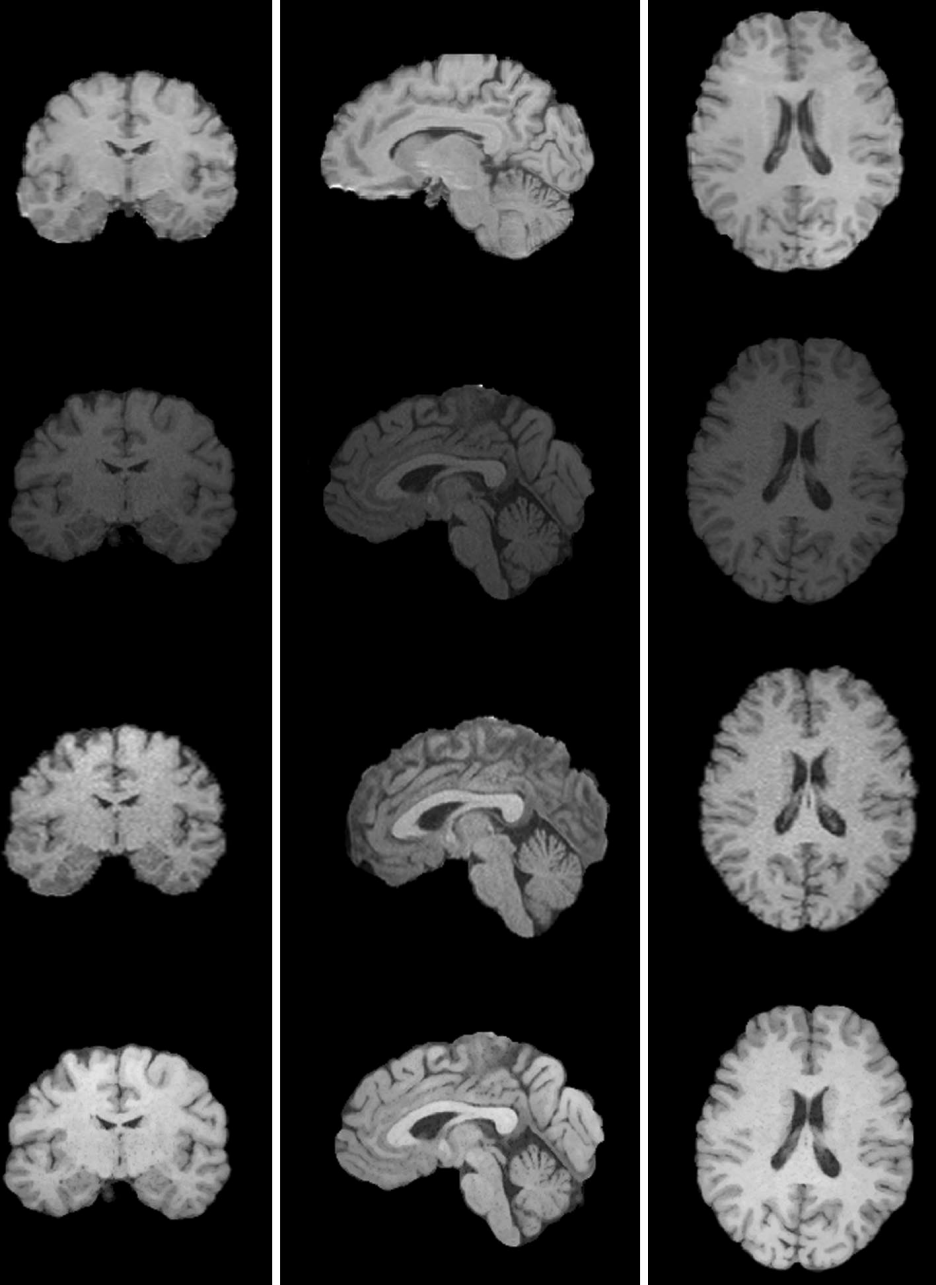
This figure shows the images from *top* to *bottom*: reference Image, input image, normalized image using histogram matching, and normalized image using histogram normalization. The quality of normalized image using histogram normalization is close to the quality of reference image, better than the normalized image using histogram matching based on a joint histogram, and gains a favorable *gray* level of the normalized image.

**Fig. 5:**
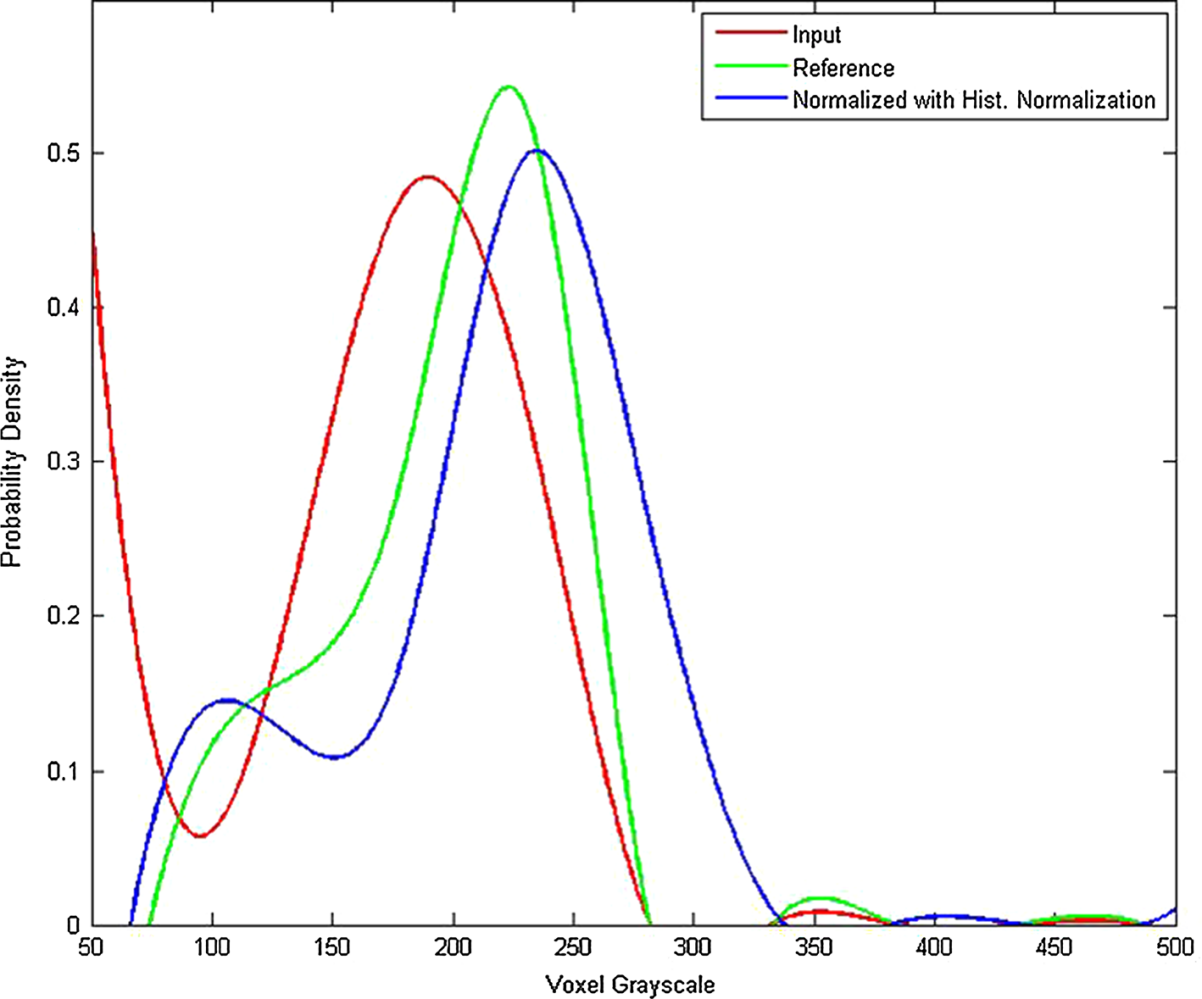
This figure shows a fitting plot of the histograms for the reference image, input image, and normalized image with histogram normalization.

As described in [6], intensity normalization results in more homogenous intensity values for voxels of the same tissue type. The qualitative effects can be seen more clearly at the image level in the visual results of registration shown in Fig. 2. After normalization and analysis, the quality of the normalized image using histogram normalization is close to the quality of the reference image, which is better than the normalized image using the histogram matching based on a joint histogram, and gains a favorable gray level for the normalized image (as shown in Fig. 4). Our method does not seek to match histograms. However, it is useful to compare them after normalization. Figure 5 shows that the histogram of one normalized image is closer to the reference histogram than the input image. We also reported on the MRI quality using an estimation index in Table 4 among the quality parameters of the input image, the reference image, and the normalized image in 11 subjects. After normalization and quality analysis, the quality of the normalized image using the histogram normalization is close to the quality of the reference image, which is better than the normalized image using the histogram matching method.

**Table 4:** Average estimation index among the quality of the input image, the reference image, and the normalized image on 11 subjects

Image type	$$ \overset{\lower0.5em\hbox{$\smash{\scriptscriptstyle\frown}$}}{\sigma }_{n} $$
Reference image	0.0026
Input image	18.0061
Normalized image with hist. matching	4.6796
Normalized image with hist. normalization	0.1675

### Chinese brain template construction

**Fig. 6:**
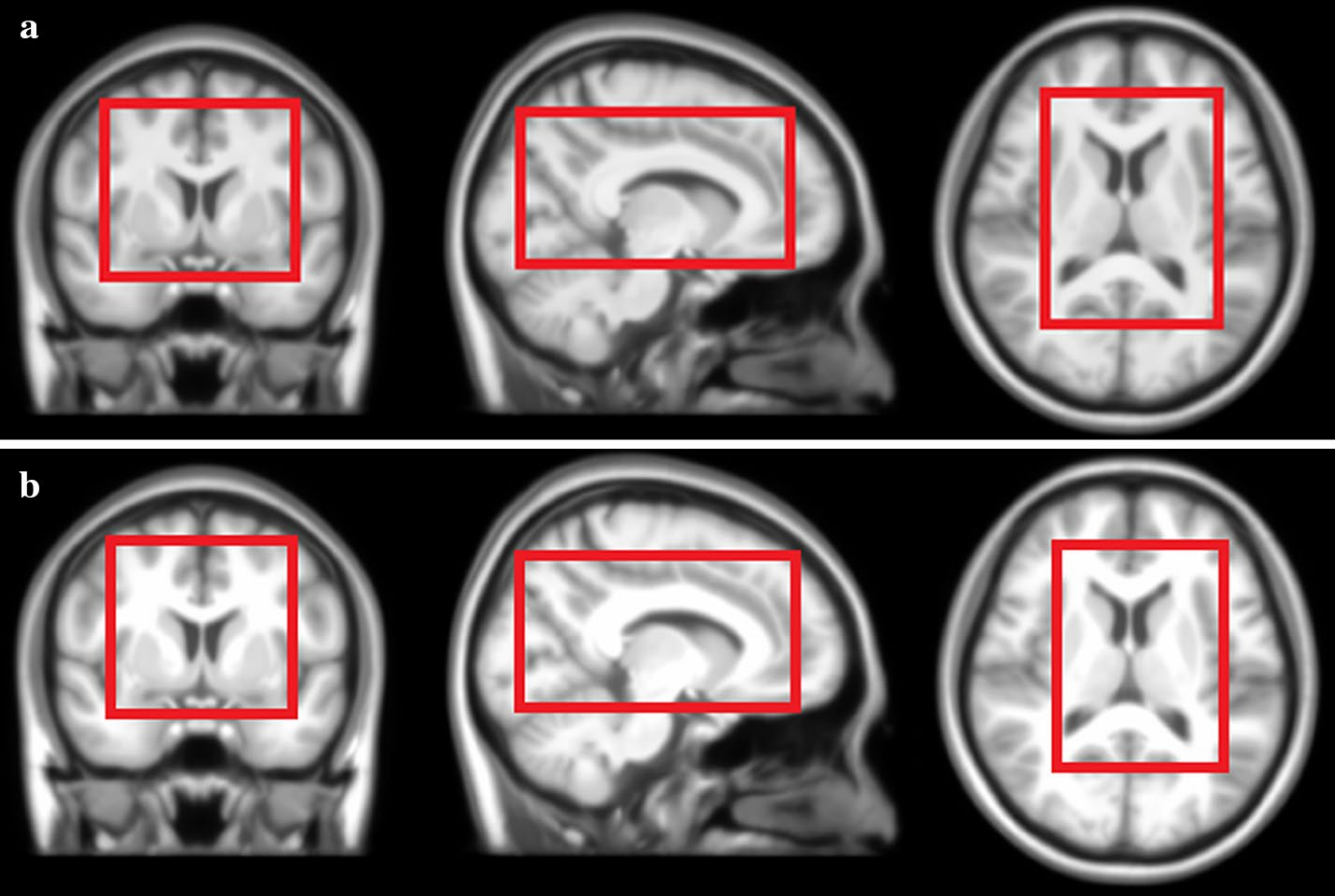
The average template of 100 Chinese adults (age ranged from 20 to 30 years) brain MR images from different scanners. **a** The average template without intensity normalization in the preprocessing procedure; **b** the average template after applying our histogram-based intensity normalization.

We constructed brain templates for Chinese young adults both with and without our normalization method. As shown in Fig. 6, Fig. 6a is the template without intensity normalization in the preprocessing procedure, and Fig. 6b is the average template after applying intensity normalization. As pointed out in the red frame, the template after intensity normalization is much clearer in some boundaries than template without intensity normalization.

## Discussion

Our results demonstrated that intensity normalization plays a very important role in improving the results of image analysis including registration, segmentation, and efficient tissues’ volumes statistics.

As shown in Fig. 2, intensity normalization through histogram normalization results in better registration results. The quantitative effects can be seen more clearly in Fig. 3 and Table 1 where the histogram normalization results in a decreased MSE between the low-quality image and the high-quality image.

In the segmentations of GM, CSF and WM, histogram normalization also results in more consistent results as indicated in the Table 2. Histogram normalization can help accurately segment different types of tissues, and contribute to many clinical applications.

Meanwhile, the tissue volume difference between the low-quality data and the high-quality data seen in Table 3 is smaller after histogram normalization of the low-quality data. One subject should have similar volumes of each tissue from the two scanners within a short time according to the clinical analysis. Histogram normalization can contribute to exactly assessing the tissues’ volumes from medical imaging, and decrease the differences of volumes in one subject to the maximum extent. This result of tissue volumes statistics further indicates that the histogram normalization can apply to normalize the different functional regions of fMRI, and assist with multi-site alignment of results in medical imaging to help clinical diagnosis and analysis.

We compare the results from three basic medical imaging processing techniques, registration, segmentation, and three brain main tissues volumes measurement before and after normalization, with our histogram normalization and the existing histogram matching method. The results again confirm the added advantage offered by the numerical approach in both absolute (increased MSE and DSC values) as well as the volumes of WM, GM and CSF.

However, a limitation of our intensity normalization technique is that intensity normalization of the MR images is performed after the whole head, fat, skull bone, and background are removed. This fact implies that the intensity normalization cannot be applied in some cases when adipose or osseous tissues may be important. Our histogram-based intensity normalization is a global normalization method. For the intensity information of local or specified tissue, it may be demonstrate that better intensity normalization in regard to local or specified tissue information. In future work, our study will solve the limit problem to do better intensity normalization of local or specified brain tissue. Apart from these considerations about the limits, our intensity normalization as a global intensity normalization scheme could be also applied in large-scale image sets acquired with different modalities, e.g. T2-weighted or proton density MR images in the future work.

## Conclusion

Tissue intensity in brain MRIs can vary remarkably due to the differences in acquisition protocols, scanner differences, heterogeneity of sources, and possibly due to intensity inhomogeneity corrections applied to obtain uniform images. As a result, intensity normalization plays a very important role in facilitating comparison between tissue intensities for various tissue types across different brain MRI volumes. Various intensity normalization procedures have been proposed to address this issue. In this work, we demonstrated the effectiveness of the histogram normalization approach across multi-scanner MRI data in the presence of various magnetic field strengths. We examined the effect of intensity normalization on the tissue intensity behavior in T1 MRIs. Improvements in image preprocessing techniques may have therefore a great impact in areas such as image analysis and computer-aided diagnosis. In the current case study of 11 patients, it was shown that our histogram normalization method can help achieve better results in image analysis compared with existing intensity normalization methods.

Our histogram normalization algorithm for brain MRI can normalize scans having the same weighting but acquired on different scanners or with different acquisition parameters. We validated our algorithm on real MRI data. Compared with the traditional histogram matching method using the joint histograms, our method showed better quality and homogeneous intensities for MR brain images after normalization. The ability of the normalization procedure to correct for global intensity variance was demonstrated through three evaluation methods. During MR image acquisition, different machines or parameters would be used for scanning different subjects or the same subject at a different time, which may result in large intensity variation across scans. On MR images of scanning different subjects, the highest-quality MR image served as the reference and was used as the standard for image quality estimation. On MR images of the same subject at a different time, the high-quality MR image from the same subject served as the reference through image quality estimation in order to perform intensity normalization on the same subject. In our method, we always guarantee the high-quality image to serve as the reference image to keep the quality of normalized images high. The histogram normalization method can be applied in different MRI sequences and imaging parts. In the future work, more MRI data, including different MRI sequences, such as T2 weight MRI, fMRI, will be acquired for further validation. Moreover, the method also be used for normalizing a large population of multi-center data and constructing the template image. Our intensity normalization was applied in the construction of a brain template of Chinese young adults (e.g. 20–30 years old adults). These results showed that intensity normalization could achieve a better image analysis performance without spatial registration. In the procedure of constructing a template of population, intensity normalization is a crucial step and has a positive impact on the final template result.
